# The Regulation and Function of the L-Type Amino Acid Transporter 1 (LAT1) in Cancer

**DOI:** 10.3390/ijms19082373

**Published:** 2018-08-12

**Authors:** Travis B Salisbury, Subha Arthur

**Affiliations:** Departments of Biomedical Sciences and Clinical & Translational Science, Joan C. Edwards School of Medicine, Marshall University, 1 John Marshall Drive, Huntington, WV 25755, USA; arthursu@marshall.edu

**Keywords:** L-Type Amino Acid Transporter 1 (LAT1), SLC7A5, mTOR, leucine, cancer

## Abstract

The progression of cancer is associated with increases in amino acid uptake by cancer cells. Upon their entry into cells through specific transporters, exogenous amino acids are used to synthesize proteins, nucleic acids and lipids and to generate ATP. The essential amino acid leucine is also important for maintaining cancer-associated signaling pathways. By upregulating amino acid transporters, cancer cells gain greater access to exogenous amino acids to support chronic proliferation, maintain metabolic pathways, and to enhance certain signal transduction pathways. Suppressing cancer growth by targeting amino acid transporters will require an in-depth understanding of how cancer cells acquire amino acids, in particular, the transporters involved and which cancer pathways are most sensitive to amino acid deprivation. L-Type Amino Acid Transporter 1 (LAT1) mediates the uptake of essential amino acids and its expression is upregulated during the progression of several cancers. We will review the upstream regulators of LAT1 and the downstream effects caused by the overexpression of LAT1 in cancer cells.

## 1. Introduction: System L Transporters

The synthesis of biomass prior to cell proliferation is heavily dependent on the acquisition of exogenous amino acids that, upon being transported into cells, are used to synthesize proteins, nucleic acids, lipids, and ATP [1]. The transporters that mediate the uptake of exogenous amino acids into cells are commonly upregulated on cancer cells compared with normal cells, and the robust acquisition of amino acids by cancer cells plays a critical role in cancer progression [2]. Many cancer cells rely on system L transporters to acquire essential amino acids [2]. The system L transporter family consists for four members: SLC7A5 (also called LAT1), SLC7A8 (also called LAT2), SLC43A1 (also called LAT3), and SLC43A2 (also called LAT4) [2]. The LAT transporters mediate the uptake of branch-chained and aromatic amino acids through a sodium-independent mechanism [2]. LAT1 and LAT2 are obligatory amino acid exchangers, and LAT3 and LAT4 mediate facilitated amino acid diffusion [2]. LAT1 and LAT2 transport leucine, isoleucine, valine, phenylalanine, methionine, tyrosine, histidine, and tryptophan into cells [2]. Leucine, isoleucine, valine, phenylalanine, and methionine can also be transported into cells by LAT3 and LAT4 [2].

In this review, we will focus on the regulation and function of LAT1 in cancer because its role in cancer is broader than the other three LAT transporters [2]. Increasing the uptake of leucine into cells is the most effective way that LAT1 promotes the activity of Mechanistic Target of Rapamycin Kinase Complex 1 (mTORC1). LAT1 not only supports mTORC1 activity, it also reinforces MYC and EZH2 signaling in cancer cells. We will review these LAT1-supported downstream effects. Maintaining a high expression and activity of LAT1 in cancer requires a large supporting cast of proteins consisting of a chaperone protein, glutamine transporters, and upstream regulators of LAT1 expression. We will review these different aspects of LAT1 regulation in cancer. Please see Table 1 for a “summative” glance of the upstream regulators of LAT1 and the downstream effects of LAT1 in cancer (Table 1).

## 2. LAT1 Promotes mTORC1 Activity

Nutrient and growth factor pathways regulate the cellular metabolism and protein synthesis by regulating the activity of mTORC1 [12]. Aberrant mTORC1 activation is common in cancer, and mTORC1 signaling promotes cancer progression by stimulating pathways that in turn support cancer cell growth, proliferation and resistance to apoptosis [12]. In regards to amino acids, leucine is the most effective activator of mTORC1 [12]. Leucine stimulates the translocation of mTORC1 to the surface of the lysosome, which is the site of mTORC1 activation [12]. The processes by which leucine regulates mTORC1 is complex because there are two leucine binding proteins and four Rag Guanosine triphosphate (GTP) binding proteins (Rag GTPases) involved [13,14,15,16]. Although there are four Rag GTPases (RagA, RagB, RagC, and RagD), RagB/RagD heterodimers are the most effective at activating mTORC1 [14]. The two leucine-binding proteins that promote the activation of mTORC1 work through different mechanisms. Leucyl-tRNA synthetase (LRS) upon binding to leucine triggers the hydrolysis of RagD-GTP to RagD-GDP, and RagD-GDP is the form that activates mTORC1 [14,16]. The binding of leucine to Sestrin2 disrupts the binding of Sestrin2 to GATOR2, which in turn allows GATOR2 to proceed with activating mTORC1 [13]. These leucine-triggered molecular events allow lysosome bound mTORC1 to carry out its signaling in response to growth factor (such as insulin or IGF1) signaling [12]. The tuberous sclerosis complex (TSC) (composed of TSC1 and TSC2) and Ras Homolog Enriched In Brain (RHEB) are insulin/IGF1 responsive regulators of mTORC1 [12]. The TSC complex promotes the GTPase activity of RHEB and inhibiting TSC activity results in GTP-loaded RHEB [12]. Insulin/IGF1-stimulated increases in PI3K/AKT signaling inhibits the TSC complex, leading to a subsequent increase in GTP-loaded RHEB and the activation of mTORC1 [12]. Consequently, LAT1 by mediating cellular uptake of exogenous leucine, supports and sustains mTORC1 activation in cancer cells.

The findings of a recent report indicate that LAT1 not only associates with the cell membrane, but it also mediates the transfer of leucine into the lysosome [17]. This requires LAT1 to be recruited to the lysosomal membrane by lysosomal-associated transmembrane protein 4b (LAPTM4b) [17]. The transfer of leucine into the lysosome mediates the activation of lysosomal membrane H^+^ ATPase (V-ATPase) [18]. V-ATPase is an additional part of the mTORC1 activating complex on the surface of the lysosome [18]. Results supporting a role for LAPTM4b in this new pathway found that its silencing reduces LAT1 association with the lysosomal membrane, decreases the uptake of leucine into the lysosome and leads to the dephosphorylation of mTORC1 target proteins [17]. The majority of these results came from studies conducted in Hela cells, and the role of LAPTM4b for mTORC1 activity was confirmed in MDA-MB-231 (breast) or HEPG2 (liver) cancer cells [17]. In addition, LAPTM4b is overexpressed in breast cancers compared with normal breast tissue, and it is more highly expressed in more advanced cancers, indicating that it is associated with breast cancer progression [19,20].

## 3. Interactions between CD98 and LAT1

LAT1 transports amino acids as a complex composed of LAT1 covalently bound to 4F2 Heavy Chain Antigen (also called CD98 and official gene name: *SLC3A2*) [2]. CD98 promotes LAT1 protein stability and mediates the translocation of LAT1 to the cell membrane [2]. Deciphering the role of CD98 in cancer is complicated because its effects could be due to enhancing amino acid signaling or integrin signaling [21]. Cormerais et al. (2016) approached this question by comparing LAT1 effects with CD98 effects in LS1747 colon cancer cells [3]. Considering the multifunctional role of CD98 in cancer, the CD98 null phenotype should be more severe than the LAT1 null phenotype. Silencing LAT1, however, was more impactful than silencing CD98 [3]. There were consequences associated with silencing CD98 in LS1747 cells. For instance, the leucine uptake by CD98 null cells was reduced (by 90%) compared with the control cells [3]. However, the reductions in the leucine uptake due to silencing CD98 did not correlate with decreases in cell proliferation [3]. Silencing CD98 in LS1747 cells did not produce smaller tumors in mice compared with the size of the control tumors [3]. This was in stark contrast to the LAT1 null LS1747 cells. Silencing LAT1 in LS1747 cells completely suppressed (by 100%) the uptake of leucine into cells [3]. This result correlated with LAT1 null LS1747 cells not being able to proliferate as rapidly as control cells [3]. LAT1 null LS174T cells produced smaller tumors in mice compared with control tumors [3].

The phosphorylation status of mTORC1 target proteins in LS1747 cells was evaluated in response to silencing LAT1 or CD98 [3]. The phosphorylation of S6K and p70 SK6 kinase was lower in LAT1 null cells compared with the control cells [3]. Conversely, the phosphorylation status of mTORC1 substrates in CD98 null cells was similar to that observed in the control cells [3]. Based on these findings, the activity of mTORC1 is dependent on LAT1, but not CD98, in LS1747 cells [3]. Increases in the phosphorylation of general control nonderepressible 2 (GCN2) and the accumulation of ATF4 protein are hallmark responses of amino acid stress, and both signals were induced in LAT1 null cells [3]. On the contrary, silencing CD98 did not affect the status of GCN2 or ATF4 compared with the control cells [3]. When taken together, finding that CD98 null cells (LS1747) maintain mTORC1 and are not amino acid-deprived is surprising, given that their leucine uptake capability was reduced by 90% compared with the control cells [3]. The remaining 10% leucine uptake capability in CD98 null cells is due to residual LAT1 activity because blocking LAT1 activity with BCH or JPH203 eliminated (100%) the leucine uptake by CD98 null cells [3]. JPH203 is also more effective at inhibiting the proliferation of CD98 null cells compared with the control cells [3]. Thus, CD98 null cells are more vulnerable to perturbation of LAT1 activity, presumably because knocking out CD98 reduced (by 90%) their capacity for leucine uptake [3]. The remaining 10% of LAT1 activity in CD98 null cells is interesting because it brings the question as to how LAT1 is transporting amino acids in the absence of CD98. Perhaps there are two LAT1 chaperone proteins in LS1747 cells.

## 4. EZH2 and LAT1 Mutual Relationship

The findings of a recent report have established reciprocal inductive interactions between LAT1 and the epigenetic regulator Enhancer of Zeste Homolog 2 (EZH2) in lung cancer cells [5]. Sorting cells by CD98 identified two populations of lung cancer cells, one population expressed high levels of CD98 and LAT1 and the other population expressed low levels of CD98 and LAT1 [5]. Cells that expressed higher levels of CD98/LAT1 exhibited a more aggressive tumor phenotype compared with cells that expressed low levels of CD98/LAT1 [5]. The CD98/LAT1 positive cells also produced larger tumors in mice compared with tumors that expressed low levels of CD98/LAT1 [5]. A metabolomic analysis identified that the levels of *S*-adenosylmethionine (SAM) were correlated with the expression of CD98/LAT1 [5]. SAM is the methyl donor used by EZH2 to tri-methylate lysine 9 on histone H3 (H3K9me3) [22]. This particular histone marker reduces tumor suppressor genes, and is one mechanism by which EZH2 is postulated to promote cancer progression [22]. Accordingly, H3K9me3 levels in the total cell extract were higher in CD98/LAT1 high cells than CD98/LAT1 low cells [5]. Whether specific EZH2 target genes were repressed in CD98/LAT1 high cells compared to CD98/LAT1 low cells was not reported, and could be a topic for future study.

The authors provided several lines of additional evidence for a link between EZH2 and LAT1 in lung cancer. Silencing and pharmacological inhibition of EZH2 in NCI-H1299 lung cancer cells caused reductions in LAT1 mRNA and protein [5]. The mechanism by which EZH2 increases LAT1 is complex because the *LAT1 gene* is not a direct EZH2 target gene [5]. In the context of retinoic acid signaling, EZH2 disinhibited LAT1 expression by reducing the expression of retinoid X receptor alpha (RXRA) [5]. The expression of LAT1 and EZH2 were also linked with more undifferentiated cancer. Promoting the differentiation of lung cancer cells by culturing them as pulmospheres caused decreases in the LAT1, CD98, and EZH2 protein levels [5]. Expressing SV40 large T-antigen in normal lung epithelial cells causes them to become cancer cells, and this correlated with the upregulation of LAT1, CD98 and EZH2 protein [5]. Within lung tumor samples, LAT1 and EZH2 were co-expressed in cancer cells that were more undifferentiated and highly proliferative compared with not being expressed in the adjacent stromal tissue [5]. Thus, LAT1 and EZH2 appear to be functionally linked with a more aggressive lung cancer phenotype. This finding has provided new insight into the regulation and function of LAT1 in cancer and inhibiting LAT1 could be a new way to suppress the cancer-promoting effects of EZH2.

## 5. LAT1 and MYC Feedforward Mechanism

The findings of a recent study identified that LAT1 and MYC amplify each other’s expression and activity in Burkitt’s lymphoma and neuroblastoma cells [6]. The authors showed that MYC induces LAT1 and LAT3, both targets of MYC, and silencing MYC decreases the level of LAT1 and LAT3 mRNA and protein [6]. Knocking down LAT1 or LAT3 expression reduced the uptake of leucine and phenylalanine into cells [6]. Accordingly, silencing MYC also reduced the uptake of leucine and phenylalanine into cells [6]. Decreases in LAT1 and LAT3 in MYC knockdown cells prevented leucine-stimulated increases in mTORC1 activity, presumably because leucine could not enter MYC null cells [6]. Silencing LAT1 inhibits the proliferation of Burkitt’s lymphoma cells (Daudi) and neuroblastoma cells (BE-2C), indicating that LAT1 induction by MYC is tied with cancer cell proliferation [6]. LAT1 null neuroblastoma tumors were smaller than control tumors in mice, indicating that LAT1 promotes the progression of this cancer [6]. Ectopic expression of LAT1 in neuroblastoma cells also produced larger tumors in mice compared with control tumors [6].

Western blot analysis of LAT1 null Burkitt’s lymphoma cells and neuroblastoma cells identified a reduction in MYC protein; however, the levels of MYC mRNA were not reduced [6]. Considering that amino acid uptake is reduced in LAT1 null cells, the observed reductions in MYC protein could reflect a defect in MYC translation due to a shortage of amino acids. Accordingly, a defect in MYC translation was confirmed in Burkitt’s lymphoma cells and neuroblastoma cells that were null for LAT1 compared with the control cells [6]. A defect in MYC translation could lead to global gene expression changes because MYC is the primary transcription factor in cancer cells. Consequently, gene expression profiling experiments were conducted in LAT1 null and MYC null cells (Burkitt’s lymphoma cells). The results indicated that the majority of gene expression changes caused by the silencing of LAT1 were likely due to the MYC protein being reduced in LAT1 null cells [6]. Specifically, silencing LAT1 changed the expression of 1120 genes [6]. Of these 1120 genes, 689 were MYC regulated genes [6]. Silencing MYC changed the expression of 3948 genes [6]. The RT-PCR analysis confirmed the downregulation of 24 MYC target genes in LAT1 knockdown cells that play roles in metabolism, translation, and growth [6].

Oncogenic MYC influences cancer cell metabolism by increasing the expression of glucose and glutamine transporters and promoting glucose and glutamine metabolism [23]. Considering that MYC translation required LAT1, the effect of LAT1 on MYC-regulated metabolic pathways was evaluated. Silencing LAT1, depriving cells of essential amino acids, or treating cells with the LAT1 inhibitor BCH (2-aminobicyclo-2,2,1-heptane-2 carboxylic acid) caused decreases in glucose and glutamine uptake by cells and reductions in aerobic glycolysis [6]. Gas chromatography–mass spectrometry analysis showed that silencing LAT1 and LAT3 affected several metabolic pathways including the TCA cycle, lipid, and nucleic acid biosynthesis pathways [6]. Collectively, finding that LAT1 reinforces oncogenic MYC signaling indicates that targeting LAT1 could be a new way to suppress MYC-driven tumors [6].

## 6. NOTCH Signaling Promotes LAT1

The NOTCH signaling pathway is an important upstream regulator of LAT1 signaling in malignant T cells [7]. The role of NOTCH in regulating LAT1 signaling during the progression of T cell leukemia was identified using a mouse model of human T-cell acute lymphoblastic leukemia (T-ALL) [7]. The mouse model involves targeted PTEN deletion from T cell progenitors in the thymus [7]. Silencing PTEN in T cells produces malignant T cells that are phenotypically similar to T-ALL in humans [7]. As expected, PTEN null T cells exhibited upregulated AKT activity [7]. The T-ALL progression correlated with robust increases in nutrient (glucose, glutamine, transferrin, and leucine) uptake by T-ALL cells compared with normal thymocytes [7]. The activated AKT was identified as not being sufficient to induce nutrient uptake by T cells, suggesting that pathways other than AKT induce the nutrient uptake by T-ALL cells [7]. Increases in mTORC1 activity, MYC, HIF1-alpha and NOTCH signaling correlated with increases in nutrient uptake by T-ALL cells [7]. Inhibiting mTORC1 activity with rapamycin reduced the levels of MYC and HIF1 alpha protein and suppressed glucose uptake by T-ALL cells [7]. Silencing HIF1 alpha in PTEN null T cells suppressed the progression of T-ALL in mice [7].

Proteomic analysis identified that levels of the LAT1 protein were higher than the levels of LAT2, LAT3 or LAT4 in T-ALL tumors from mice [7]. Silencing LAT1 in T-ALL tumors improved the survival of mice compared with mice with LAT1 expressing T-ALL tumors [7]. Western blot analysis identified reduced mTORC1 activity in LAT1 null T-ALL tumors compared with wild-type T-ALL tumors [7]. In terms of signaling, the NOTCH pathway was identified as being important for LAT1 expression in T-ALL cells. For instance, the NOTCH ligand delta-like 1 (DL1) simulated leucine uptake by normal T lymphocytes [7]. Antagonizing LAT1 suppressed leucine uptake by T cells in response to DL1 [7]. Inhibiting NOTCH in T-ALL cells with a gamma-secretase inhibitor suppressed the leucine uptake by T-ALL cells [7]. Suppressing the NOTCH signaling not only suppressed leucine uptake, it also inhibited glucose uptake by T-ALL cells [7]. Finding that NOTCH supports leucine uptake by T-ALL cells indicates that inhibiting NOTCH could be a new way to suppress leucine signaling to inhibit T-ALL.

## 7. LAT1 and AHR

LAT1 is overexpressed in breast cancer compared with normal breast tissue, and it is more highly expressed in more advanced cancers, indicating that it is associated with breast cancer progression [24,25]. We have recently reported that LAT1 is an aryl hydrocarbon receptor (AHR) target gene and that reducing AHR or LAT1 with short interfering RNA (siRNA) reduces the proliferation of MCF7 and MDA-MB-231 breast cancer cells [9]. Dioxins are widespread environmental toxicants that bind AHR with high affinity. Dioxins are tumor promoting or tumor suppressing depending on the cancer type and the timing of dioxin exposure [26]. The compound 2,3,7,8-Tetrachlorodibenzo-p-dioxin (TCDD) binds to AHR with high affinity and it is the prototype dioxin. TCDD induces the binding of an AHR complex to intron 2 of the *LAT1* gene that consists of the AHR, AHR nuclear translocator (ARNT) and the transcriptional coactivator p300 [9]. TCDD-stimulated increases in LAT1 transcription led to increases in leucine uptake by MCF7 breast cancer cells [9]. In addition to being responsive to exogenous ligands, the AHR has a constitutive activity that modulates gene expression in breast cancer [27]. In this regard, silencing AHR (in the absence of exogenous AHR ligands) in MCF7 and MDA-MB-231 cells decreased the LAT1 mRNA and LAT1 protein, indicating that constitutive AHR activity promotes the transcription of LAT1 in these breast cancer models [9]. These results suggest that the regulation of LAT1 by AHR in breast cancer could be important for the progression of this disease.

## 8. The Role of ATF4 and LAT1 in Prostate Cancer

LAT1 and LAT3 play important roles at different stages of prostate cancer progression. When prostate tumors are responsive to androgen, LAT3 is the dominant leucine transporter because the *LAT3* gene is a direct androgen target gene [10]. As prostate tumors transition from being hormone responsive to hormone refractory, the expression of LAT3 decreases and the expression of LAT1 increases [10]. The upregulation of LAT1 in hormone-resistant prostate tumors is due to its transcriptional activation by ATF4 [10]. Hormone refractory prostate tumors express high levels of ATF4 because they have responded to the amino acid shortage caused by the loss of LAT3 [10]. In prostate cancer, LAT1 and LAT3 not only contribute to tumor bulk by mediating cancer cell proliferation, they also support metastasis [11]. Indeed, LAT1 or LAT3 null prostate tumors (PC-3) were smaller and they were less metastatic than wild-type tumors in mice [11]. Gene expression profiling experiments in prostate cancer cells showed that LAT1- and LAT3-regulated genes were highly associated with cell cycle progression, E2F transcription factors and high-grade metastatic prostate cancer [11]. Collectively, these results show that LAT1 expression is associated with hormone-resistant prostate cancer and that its expression is induced by ATF4 in this disease [10,11].

## 9. The Role of Glutamine in Supporting LAT1 Activity

Work conducted nearly 10 years ago showed that the cellular uptake of glutamine and leucine activated mTORC1 and suppressed autophagy in Hela cells [28]. The authors found that glutamine did not activate mTORC1 through a direct mechanism [28]. Instead, glutamine was transported into Hela cells by SLC1A5, which indirectly supported mTORC1 activity by inducing the activity of LAT1 [28]. Intracellular glutamine induces LAT1 activity; because LAT1 is an obligate amino acid exchanger that must transport an amino acid out of the cell as it transports leucine into the cell [28]. In Hela cells, glutamine is the required efflux substrate for LAT1 that induces the LAT1-mediated uptake of leucine [28]. Glutamine-free cell culture media or preventing glutamine uptake into Hela cells by silencing SLC1A5 inhibits the LAT1-mediated uptake of leucine [28]. Reductions in LAT1 activity caused concomitant reductions in mTORC1 activity, which in turn increased autophagy [28].

SLC1A5 maintains LAT1 activity in Hela cells, but not in all cell types. For instance, knocking out SLC1A5 from A549 (lung) or LS174T (colon) cancer cells inhibited glutamine uptake into cells by approximately 70% without inhibiting leucine uptake into cells by LAT1 [4]. In A549 and LS174T cells, knocking out LAT1 reduced the mTORC1 activity and induced the amino acid stress pathway; however, knocking out SLC1A5 did not affect the activity of these two amino acid responsive pathways [4]. LAT1 and SLC1A5 are required for the growth of A549 and LS174T tumors in mice [3,4]. Western blot analysis of LAT1 null tumors revealed the suppression of mTORC1 signaling and activation of the amino acid stress pathway [3]. Reductions in mTORC1 activity and increases in the amino acid stress pathway were not evident in the SLC1A5 tumors [4]. Collectively, these findings indicated that LAT1 and SLC1A5 are important for the growth of lung and colon tumors in mice; however, they support the growth of tumors through different mechanisms [3,4]. Mechanistically, LAT1 promoted tumor growth by supporting mTORC1 activity and preventing the activation of amino acid stress pathway [3]. The mechanism by which SLC1A5 supported tumor growth does not involve the mTORC1 pathway [4]. Similar findings were reported in the osteosarcoma 143B cell line, showing that SLC1A5 knockout decreased glutamine transport (by ~60%) without affecting the mTORC1 activity or cell proliferation [29]. With respect to the osteosarcoma cell study, the authors proposed that glutamine transported into cells by sodium-neutral-amino-acid transporters (SNAT) 1 and 2 maintained mTORC1 and LAT1 activity [29].

## 10. LAT1 Inhibitor JPH203

As noted in a previous section, T-ALL in mice is caused by rendering thymic T cells null for PTEN. Gene expression studies identified that the expression of LAT1 increases during the progression of T-ALL in mice [8]. T-ALL mouse cell lines also exhibited increases in LAT1 expression compared with normal T cells [8]. The higher expression of CD98, which is the LAT1 chaperone protein, was observed in patient-derived T-ALL cells compared with resting or activated peripheral blood lymphocytes from normal patients [8]. Analyzing the gene expression changes in patient samples (*n* = 72) confirmed that the expression of LAT1 was higher in T-ALL cells compared with normal T lymphocytes [8].

LAT1 inhibitors decrease the proliferation and viability of T-ALL cells [8]. Three different LAT1 inhibitors were tested: BCH, d-leucine, and JPH203 [8]. Of the three, JPH203 is the most selective LAT1 inhibitor [8]. JPH203 was also the most potent inhibitor of T-ALL cell viability [8]. JPH203 reduced the viability of mouse and patient-derived T-ALL cells growing in a cell culture, without being toxic to normal T cells (mouse or human) [8]. JPH203 also suppressed T-ALL tumor growth in mice [8]. JPH203 did not affect the viability of normal T cells or other types of blood cells (erythrocytes, platelets, bone marrow progenitors) [8]. Mice dosed with JPH203 did not exhibit reductions in body weight compared with mice dosed with the vehicle [8]. The differentiation of normal thymocytes was not disrupted by JPH203 [8]. The observed selectivity of JPH203 towards T-ALL cells is presumably because the expression of LAT1 was higher on T-ALL cells compared with normal T cells [8].

In T-ALL cells, JPH203 induces several signaling pathways [8]. JPH203 was a strong inducer of the C/EBP homologous protein (CHOP) and the unfolded protein response (UPR) [8]. CHOP is induced in response to endoplasmic reticulum (ER) stress and it promotes apoptosis [30]. The JPH203 induction of CHOP and UPR was rapid (within 2 h of treatment) and it occurred before the other JPH203 effects [8]. The other JPH203 effects occurring after the induction of CHOP were increases in apoptosis, autophagy, and decreases in mTORC1 activation and MYC protein levels [8]. Starving T-ALL cells of amino acids was sufficient to induce CHOP, which is consistent with how JPH203 would induce CHOP [8]. T-ALL cells treated with the UPR inhibitor salubrinal were resistant to JPH203-stimulated increases in CHOP [8]. Salubrinal also restored proliferation, increased cell viability and suppressed apoptosis of T-ALL cells treated with JPH203 [8]. Based on these results, the authors proposed that JHP203 induces T-ALL cell death via the induction of CHOP and UPR [8]. In support of this postulate, JPH203 fails to induce CHOP in a JHP203 resistant T-ALL cell line [8].

The authors also tested how combining JPH203 with other cancer drugs affected T-ALL survival in cell culture [8]. The findings showed that JPH203 interacted synergistically (strongly) with the mTOR inhibitor rapamycin [8]. Its interaction with dexamethasone ranged from being primarily synergistic, but also additive, depending on the drug concentration [8]. When combined with KU0063794 (a dual mTORC1, mTORC2 inhibitor), PI103 (PI3K inhibitor), doxorubicin or bortezomib (proteasome inhibitor) its effects were drug and concentration-dependent and ranged from antagonism to synergistic [8]. Finally, the effect of combining JPH203 with l-asparaginase ranged from antagonism (at high drug concentrations), to additive (at middle drug concentrations), to highly synergistic (lowest drug concentrations) [8]. The effect of JPH203 on other cancer cell types have been tested, and the reported effects have shown that it inhibits the cell cycle and/or promoted apoptosis in cancer cells. Specifically, JPH203 inhibits osteosarcoma [31], cholangiocarcinoma [32], human thymic carcinoma cells [33], and lung cancer cells [3]. Thus, these reports indicate that JPH203 anti-cancer effects could be applicable to other cancers that express LAT1 and that it is not toxic toward normal cells.

## 11. Conclusions

From all apparent evidence, the LAT1/amino acid signaling pathway in cancer cells is an effective target for cancer therapy. There is still a need for uncovering new ways to target the cancer-specific expression of LAT1, given that LAT1 is important for proper brain function and for the expansion of normal T cells [34,35]. The possibility that targeting LAT1 stimulates the compensatory upregulation of a different LAT transporter (i.e., LAT2, LAT3, or LAT4), or a different transporter system (i.e., SNAT amino acid transporters [29]) in certain cancers also warrants investigation. There is also a need to identify specific biomarkers to predict sensitivity or resistance to LAT1 targeting therapies. Thus, collectively, the LAT1-leucine-signaling pathway in cancer and its therapeutic targeting is still a very active area of research.

## Figures and Tables

**Table 1:** The cancer-specific regulation and function of L-Type Amino Acid Transporter 1 (LAT1).

Cancer Type	Cell Line(s)	Upstream Regulators of LAT1	Downstream Effects of LAT1	References
Lung, Colon	A549, LS174T	N/A	LAT1 silencing led to smaller tumors in mice, reductions in leucine uptake, reductions in mTORC1 activity, amino acid stress, decreases in proliferation	[3,4]
Lung	NCI-H1299	EZH2	LAT1 silencing led to smaller tumors in mice, decreases in EZH2 expression and activity	[5]
Glioblastoma, Burkitt’s Lymphoma	Daudi, BE-2C, SHEP MYCN-ER, P493	MYC	LAT1 silencing led to reductions in MYC translation, decreases in leucine uptake, smaller tumors in mice, decreases in proliferation, altered cancer cell metabolism, many gene expression changes	[6]
T-cell acute lymphoblastic leukemia (T-ALL)	Mouse model of T-ALL, patient-derived T-ALL cells	NOTCH	LAT1 silencing led to the inhibition of T-ALL progression in vivo	[7]
T-cell acute lymphoblastic leukemia (T-ALL)	Mouse model of T-ALL, patient-derived T-ALL cells	JPH203 (LAT1 antagonist)	LAT1 antagonism by JPH203 led to decreases in proliferation, increases in apoptosis, smaller T-ALL tumors in mice, increases in CHOP and ER stress, apoptosis, and autophagy and decreases in mTORC1 activity	[8]
Breast	MDA-MB-231, MCF7	TCDD, AHR	LAT1 silencing led to decreases in proliferation	[9]
Prostate	PC-3	ATF4	LAT1 silencing led to smaller tumors in mice, decreases in metastasis in mice, reductions in leucine uptake, reductions in mTORC1 activity, amino acid stress, decreases in proliferation, increases in apoptosis, many gene expression changes	[10,11]

## References

[1] Hosios A.M., Hecht V.C., Danai L.V., Johnson M.O., Rathmell J.C., Steinhauser M.L., Manalis S.R., Vander Heiden M.G. (2016). Amino Acids Rather than Glucose Account for the Majority of Cell Mass in Proliferating Mammalian Cells. Dev. Cell.

[2] Wang Q., Holst J. (2015). L-type amino acid transport and cancer: Targeting the mTORC1 pathway to inhibit neoplasia. Am. J. Cancer Res..

[3] Cormerais Y., Giuliano S., LeFloch R., Front B., Durivault J., Tambutte E., Massard P.A., de la Ballina L.R., Endou H., Wempe M.F. (2016). Genetic Disruption of the Multifunctional CD98/LAT1 Complex Demonstrates the Key Role of Essential Amino Acid Transport in the Control of mTORC1 and Tumor Growth. Cancer Res..

[4] Cormerais Y., Massard P.A., Vucetic M., Giuliano S., Tambutte E., Durivault J., Vial V., Endou H., Wempe M.F., Parks S.K. (2018). The glutamine transporter ASCT2 (SLC1A5) promotes tumor growth independently of the amino acid transporter LAT1 (SLC7A5). J. Biol. Chem..

[5] Dann S.G., Ryskin M., Barsotti A.M., Golas J., Shi C., Miranda M., Hosselet C., Lemon L., Lucas J., Karnoub M. (2015). Reciprocal regulation of amino acid import and epigenetic state through Lat1 and EZH2. EMBO J..

[6] Yue M., Jiang J., Gao P., Liu H., Qing G. (2017). Oncogenic MYC Activates a Feedforward Regulatory Loop Promoting Essential Amino Acid Metabolism and Tumorigenesis. Cell Rep..

[7] Grzes K.M., Swamy M., Hukelmann J.L., Emslie E., Sinclair L.V., Cantrell D.A. (2017). Control of amino acid transport coordinates metabolic reprogramming in T-cell malignancy. Leukemia.

[8] Rosilio C., Nebout M., Imbert V., Griessinger E., Neffati Z., Benadiba J., Hagenbeek T., Spits H., Reverso J., Ambrosetti D. (2015). L-type amino-acid transporter 1 (LAT1): A therapeutic target supporting growth and survival of T-cell lymphoblastic lymphoma/T-cell acute lymphoblastic leukemia. Leukemia.

[9] Tomblin J.K., Arthur S., Primerano D.A., Chaudhry A.R., Fan J., Denvir J., Salisbury T.B. (2016). Aryl hydrocarbon receptor (AHR) regulation of L-Type Amino Acid Transporter 1 (LAT-1) expression in MCF-7 and MDA-MB-231 breast cancer cells. Biochem. Pharmacol..

[10] Wang Q., Bailey C.G., Ng C., Tiffen J., Thoeng A., Minhas V., Lehman M.L., Hendy S.C., Buchanan G., Nelson C.C. (2011). Androgen receptor and nutrient signaling pathways coordinate the demand for increased amino acid transport during prostate cancer progression. Cancer Res..

[11] Wang Q., Tiffen J., Bailey C.G., Lehman M.L., Ritchie W., Fazli L., Metierre C., Feng Y.J., Li E., Gleave M. (2013). Targeting amino acid transport in metastatic castration-resistant prostate cancer: Effects on cell cycle, cell growth, and tumor development. J. Natl. Cancer Inst..

[12] Saxton R.A., Sabatini D.M. (2017). mTOR Signaling in Growth, Metabolism, and Disease. Cell.

[13] Wolfson R.L., Chantranupong L., Saxton R.A., Shen K., Scaria S.M., Cantor J.R., Sabatini D.M. (2016). Sestrin2 is a leucine sensor for the mTORC1 pathway. Science (New York, N.Y.).

[14] Lee M., Kim J.H., Yoon I., Lee C., Fallahi Sichani M., Kang J.S., Kang J., Guo M., Lee K.Y., Han G. (2018). Coordination of the leucine-sensing Rag GTPase cycle by leucyl-tRNA synthetase in the mTORC1 signaling pathway. Proc. Natl. Acad. Sci. USA.

[15] Sancak Y., Bar-Peled L., Zoncu R., Markhard A.L., Nada S., Sabatini D.M. (2010). Ragulator-Rag complex targets mTORC1 to the lysosomal surface and is necessary for its activation by amino acids. Cell.

[16] Han J.M., Jeong S.J., Park M.C., Kim G., Kwon N.H., Kim H.K., Ha S.H., Ryu S.H., Kim S. (2012). Leucyl-tRNA synthetase is an intracellular leucine sensor for the mTORC1-signaling pathway. Cell.

[17] Milkereit R., Persaud A., Vanoaica L., Guetg A., Verrey F., Rotin D. (2015). LAPTM4b recruits the LAT1-4F2hc Leu transporter to lysosomes and promotes mTORC1 activation. Nat. Commun..

[18] Zoncu R., Bar-Peled L., Efeyan A., Wang S., Sancak Y., Sabatini D.M. (2011). mTORC1 senses lysosomal amino acids through an inside-out mechanism that requires the vacuolar H(+)-ATPase. Science (New York, N.Y.).

[19] Li S., Wang L., Meng Y., Chang Y., Xu J., Zhang Q. (2017). Increased levels of LAPTM4B, VEGF and survivin are correlated with tumor progression and poor prognosis in breast cancer patients. Oncotarget.

[20] Xiao M., Yang S., Meng F., Qin Y., Yang Y., Jia S., Cai X., Li C., Huang Y., Ning X. (2017). LAPTM4B Predicts Axillary Lymph Node Metastasis in Breast Cancer and Promotes Breast Cancer Cell Aggressiveness in Vitro. Cell. Physiol. Biochem. Int. J. Exp. Cell. Physiol. Biochem. Pharmacol..

[21] Ip H., Sethi T. (2016). CD98 signals controlling tumorigenesis. Int. J. Biochem. Cell Biol..

[22] Han Li C., Chen Y. (2015). Targeting EZH2 for cancer therapy: Progress and perspective. Curr. Protein Pept. Sci..

[23] Goetzman E.S., Prochownik E.V. (2018). The Role for Myc in Coordinating Glycolysis, Oxidative Phosphorylation, Glutaminolysis, and Fatty Acid Metabolism in Normal and Neoplastic Tissues. Front. Endocrinol..

[24] El Ansari R., Craze M.L., Miligy I., Diez-Rodriguez M., Nolan C.C., Ellis I.O., Rakha E.A., Green A.R. (2018). The amino acid transporter SLC7A5 confers a poor prognosis in the highly proliferative breast cancer subtypes and is a key therapeutic target in luminal B tumours. Breast Cancer Res. BCR.

[25] Bartlett J.M., Thomas J., Ross D.T., Seitz R.S., Ring B.Z., Beck R.A., Pedersen H.C., Munro A., Kunkler I.H., Campbell F.M. (2010). Mammostrat as a tool to stratify breast cancer patients at risk of recurrence during endocrine therapy. Breast Cancer Res. BCR.

[26] Safe S., Lee S.O., Jin U.H. (2013). Role of the aryl hydrocarbon receptor in carcinogenesis and potential as a drug target. Toxicol. Sci. An Off. J. Soc. Toxicol..

[27] Salisbury T.B., Tomblin J.K., Primerano D.A., Boskovic G., Fan J., Mehmi I., Fletcher J., Santanam N., Hurn E., Morris G.Z. (2014). Endogenous aryl hydrocarbon receptor promotes basal and inducible expression of tumor necrosis factor target genes in MCF-7 cancer cells. Biochem. Pharmacol..

[28] Nicklin P., Bergman P., Zhang B., Triantafellow E., Wang H., Nyfeler B., Yang H., Hild M., Kung C., Wilson C. (2009). Bidirectional transport of amino acids regulates mTOR and autophagy. Cell.

[29] Broer A., Rahimi F., Broer S. (2016). Deletion of Amino Acid Transporter ASCT2 (SLC1A5) Reveals an Essential Role for Transporters SNAT1 (SLC38A1) and SNAT2 (SLC38A2) to Sustain Glutaminolysis in Cancer Cells. J. Biol. Chem..

[30] Yang Y., Liu L., Naik I., Braunstein Z., Zhong J., Ren B. (2017). Transcription Factor C/EBP Homologous Protein in Health and Diseases. Front. Immunol..

[31] Choi D.W., Kim D.K., Kanai Y., Wempe M.F., Endou H., Kim J.K. (2017). JPH203, a selective L-type amino acid transporter 1 inhibitor, induces mitochondria-dependent apoptosis in Saos2 human osteosarcoma cells. Korean J. Physiol. Pharmacol. Off. J. Korean Physiol. Soc. Korean Soc. Pharmacol..

[32] Yothaisong S., Dokduang H., Anzai N., Hayashi K., Namwat N., Yongvanit P., Sangkhamanon S., Jutabha P., Endou H., Loilome W. (2017). Inhibition of l-type amino acid transporter 1 activity as a new therapeutic target for cholangiocarcinoma treatment. Tumour Biol. J. Int. Soc. Oncodev. Biol. Med..

[33] Hayashi K., Jutabha P., Maeda S., Supak Y., Ouchi M., Endou H., Fujita T., Chida M., Anzai N. (2016). LAT1 acts as a crucial transporter of amino acids in human thymic carcinoma cells. J. Pharmacol. Sci..

[34] Tarlungeanu D.C., Deliu E., Dotter C.P., Kara M., Janiesch P.C., Scalise M., Galluccio M., Tesulov M., Morelli E., Sonmez F.M. (2016). Impaired Amino Acid Transport at the Blood Brain Barrier Is a Cause of Autism Spectrum Disorder. Cell.

[35] Sinclair L.V., Rolf J., Emslie E., Shi Y.B., Taylor P.M., Cantrell D.A. (2013). Control of amino-acid transport by antigen receptors coordinates the metabolic reprogramming essential for T cell differentiation. Nat. Immunol..

